# Genome-Wide Identification and Variation Analysis of *JAZ* Family Reveals *BnaJAZ8.C03* Involved in the Resistance to *Plasmodiophora brassicae* in *Brassica napus*

**DOI:** 10.3390/ijms232112862

**Published:** 2022-10-25

**Authors:** Lixia Li, Gaoxiang Ji, Wenjie Guan, Fang Qian, Hao Li, Guangqin Cai, Xiaoming Wu

**Affiliations:** Key Laboratory of Biology and Genetic Improvement of Oil Crops, Ministry of Agriculture and Rural Affairs, Oil Crop Research Institute, Chinese Academy of Agricultural Sciences, Wuhan 430062, China

**Keywords:** *Brassica napus*, JAZ, phylogenetic analysis, *Plasmodiophora brassicae*, structural variation

## Abstract

Clubroot caused by *Plasmodiophora brassicae* led to a significant decrease in the yield and quality of *Brassica napus*, one of the most important oil crops in the world. JAZ proteins are an essential repressor of jasmonates (JAs) signaling cascades, which have been reported to regulate the resistance to *P. brassicae* in *B. napus*. In this study, we identified 51, 25 and 26 JAZ proteins in *B. napus*, *B. rapa* and *B. oleracea*, respectively. Phylogenetic analysis displayed that the notedJAZ proteins were divided into six groups. The JAZ proteins clustered in the same group shared a similar motif composition and distribution order. The 51 *BnaJAZs* were not evenly assigned on seventeen chromosomes in *B. napus*, except for A04 and C07. The *BnaJAZs* of the *AtJAZ7*/*AtJAZ8* group presented themselves to be significantly up-regulated after inoculation by *P. brassicae*. Variation analysis in a population with a specific resistance performance in *P. brassicae* displayed a 64 bp translocation in *BnaC03T0663300ZS* (*BnaJAZ8.C03*, homologous to *AtJAZ8*) with an 8% reduction in the disease index on average. Through protein–protein interaction analysis, 65 genes were identified that might be involved in *JAZ8* regulation of resistance to *P. brassicae* in *B. napus*, which provided new clues for understanding the resistance mechanism to *P. brassicae*.

## 1. Introduction

Jasmonates (JAs) are one of the important defense-signaling hormones [1], which are derived from α-linoleic acid released by the oxidation of membrane lipids [2]. The main bioactive form of JAs, JA-Ile, is perceived to ultimately trigger a set of transcriptional reprogramming and produce specialized metabolites or proteins in response to abiotic/biotic stresses [3,4]. The core JAs signaling pathway constitutes several functional modules, including a repertoire of COI1–JAZ (Coronatine Insensitive1–Jasmonate-Zim Domain) co-receptors [5]. COI1 is identified to encode an F-box protein [6], which provides substrate specificity for the Skp–Cullin–F-box-type E3 ubiquitin ligase complex (SCF^COI1^) [7]. JAZ proteins target SCF^COI1^ [8], which plays a central role within the signaling cascades triggered by JAs. In plant cells with a low concentration of JAs, the expression of JA early response genes is inhibited by JAZ proteins binding to transcription factors (TFs) or an adaptor protein NINJA [9]. When external stimuli stimulate plants, the content of JAs in plants increases, accelerating the binding of SCF^COI1^ with JAZ protein and JAZ ubiquitination. As a result, the JAZ proteins are degraded by 26S proteasome, releasing TFs inhibited by JAZ proteins and finally activating the expression of JA early response genes [10].

JAZ proteins are the plant-specific zinc finger proteins belonging to the superfamily of TIFY [11]. The JAZ proteins contain a highly conserved TIF[F/Y]XG motif within the ZIM domain (also known as TIFY domain) near its N-terminal, which mediates the interaction of proteins [12,13,14]. JAZ proteins also contain a conserved Jas domain (also known as CCT_2 domain) composed of 12–29 amino acids (SLX2FX2KRX2RX5PY) near its C-terminal [15], a variant of CCT motif. The Jas domain plays a dual role in JAs signaling pathway, mediates the interaction between JAZ and its downstream TFs, and promotes the combination with COI1 [16]. The JAZ proteins lacking a Jas sequence showed JA-dominant insensitivity [17]. The *JAZ* family has large numbers of genes. Previously, the 15, 14 and 16 *JAZ* genes were found in rice [18], wheat [19] and maize [20], respectively. In *Arabidopsis thaliana*, 13 *JAZ* genes have been reported. Among them, *AtJAZ1*-*12* encodes typical TIFY JAZ proteins, which conserve the ZIM and TIFY domains. *AtJAZ13* encodes a non-TIFY JAZ protein that contains divergent ZIM, Jas and EAR motifs. Compared with other JAZ proteins, AtJAZ13 has a ser-rich C-terminal tail for phosphorylation [21]. The identified *JAZ* genes have been proven to participate in plant development and defense reactions. For example, *AtJAZ2* prevents pathogen penetration by triggering stomata closure [22]. The overexpression *JAZ2* from *Glycine soja* could enhance tolerance to salt and alkali stresses [23]. In addition, the overexpression *JAZ4* from grapes could improve the resistance to powdery mildew [24]. The expression of *JAZ1* and *JAZ5* could be affected by *WRKY57*, which compromised the resistance to *Botrytis cinerea* in *Arabidopsis* [25]. In addition, *OsJAZ9* has been reported to regulate salt stress or potassium deficiency tolerance in rice [26]. *AtJAZ10* was induced by wounding and negatively regulated the disease symptom development during *Pseudomonas syringae* pathogenesis [27].

*Brassica napus* (AACC) is one of the most important oil crops in China and the world, whose safety production is very important for supplying edible vegetable oil. However, clubroot, caused by *Plasmodiophora brassicae*, has rapidly spread worldwide, resulting in significant decreases in yield and quality in *B. napus* that result in significant economic losses yearly [28].

Breeding varieties with durable and stable resistance is the most economical and effective method to prevent clubroot. Moreover, identifying genes and regulatory pathways is the key to the genetic improvement of resistance to *P. brassicae*. In a previous study, we found that JAZ proteins, which are the repressors of JAs signaling pathway, are involved in the regulation of resistance to *P. brassicae* through compared transcriptome analysis in *B. napus* [29]. Even so, there are many members of the JAZ family in *B. napus*. It is unknown which JAZ proteins are involved in the regulation of resistance to *P. brassicae* in *B. napus* exactly. We focused on the *JAZ* genes in this study and performed genome-wide identification in *B. napus* using bioinformatics analysis. Furthermore, we analyzed the chromosome location and structure characteristics. Then, phylogenetic analysis of JAZs among *B. rapa*, *B. oleracea* and *B. napus* was conducted. In addition, expression levels of *BnaJAZs* in response to *P. brassicae* and variations of differential expressional *BnaJAZs* in the population were analyzed. The results of this study could provide critical clues for us to understand the mechanism of resistance to *P. brassicae* in *B. napus*.

## 2. Results

### 2.1. Genome-Wide Identification of JAZ in B. napus, B. rapa and B. oleracea

To identify all the JAZ proteins in *B. napus*, *B. rapa* and *B. oleracea*, we performed a Blastp and HMMER search simultaneously against the protein sequences of *B. napus* (ZS11), *B. rapa* (Chiifu V3.0) and *B. oleracea* (JZS V2.0). The merged candidate proteins were further analyzed using NCBI-CDD and SMART to ascertain the integrity of the conserved domains. Consequently, we identified 51, 25 and 26 JAZs in *B. napus*, *B. rapa* and *B. oleracea*, respectively. The amino acid (aa) residues of all the 102 JAZs ranged from 116 to 573, with an average length of 258 aa. The molecular weight of these JAZs varied from 13.1 to 62.8 kDa. In addition, the isoelectric point values were distributed from 4.96 to 10.07. The negative GRAVY scores indicated that all the JAZs were hydrophilic proteins. Subcellular localization showed that all the JAZs were predicted in the nuclei. It is worth noting that besides the nucleus, BnaC03T0060300ZS and BnaA03T0052500ZS were also found in the cell membrane or chloroplast (Table S2), which may affect multi-located proteins.

### 2.2. Phylogenetic Analysis of JAZ Family among Arabidopsis, B. napus, B. rapa, and B. oleracea

**Figure 1:**
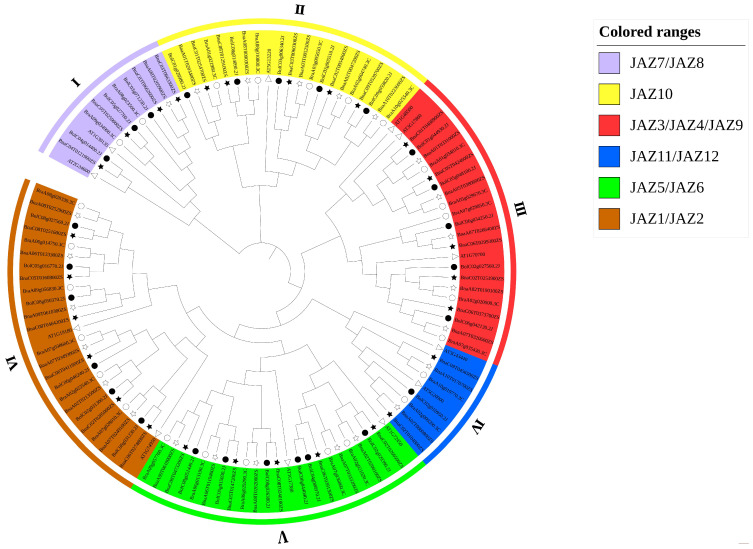
Phylogenetic analysis of 114 JAZ proteins from *Arabidopsis*, *B. napus*, *B. rapa*, and *B. oleracea*. The JAZ proteins from *B. napus* (51), *B. rapa* (25), *B. oleracea* (26), and *Arabidopsis* (12) are marked with stars, hollow circles, solid circles, and triangles, respectively. The number of bootstrap values was based on 1000 iterations. The hollow and solid stars represent genes located in the A and C subgenomes in *B. napus*, respectively. The neighbor-joining (NJ) tree shows six groups marked by Roman numerals and different colors: group I (purple) homologous to AtJAZ7/AtJAZ8, group II (yellow) homologous to AtJAZ10, group III (red) homologous to AtJAZ3/AtJAZ4/AtJAZ9, group IV (blue) homologous to AtJAZ11/AtJAZ12, group V (green) homologous to AtJAZ5/AtJAZ6 and group VI (orange) homologous to AtJAZ1/AtJAZ2.

Based on the JAZ protein sequences from *Arabidopsis*, *B. napus*, *B. rapa* and *B. oleracea*, a neighbor-joining (NJ) phylogenetic tree was constructed using MEGA11 software (Figure 1). The phylogenetic analysis revealed that all the JAZs could be divided into six groups: group I (purple, homologous to AtJAZ7/AtJAZ8), group II (yellow, homologous to AtJAZ10), group III (red, homologous to AtJAZ3/AtJAZ4/AtJAZ9), group IV (blue, homologous to AtJAZ11/AtJAZ12), group V (green, homologous to AtJAZ5/AtJAZ6), and group VI (orange, homologous to AtJAZ1/AtJAZ2). The tree showed that group VI contained the maximal JAZ members (24 proteins), while group IV had the least members (seven proteins). We found that the number of JAZ in *B. napus* was equal to the total of *B. oleracea* and *B. rapa* in most groups. Nevertheless, in group V, the total number of JAZs in *B. oleracea* and *B. rapa* was more than that in *B. napus*. Furthermore, we found the numbers of JAZs on the A genome from *B. rapa*, and C genome from *B. oleracea* were equal to that on A-subgenome and C-subgenome from *B. napus,* respectively, in all the groups except for the group I, IV, and V. Furthermore, AtJAZ1, AtJAZ2, AtJAZ5, and AtJAZ9 had six copies in *B. napus*, and also had three copies in both *B. rapa* and *B. oleracea*. Similarly, AtJAZ3 had four copies in *B. napus* and two copies in *B. rapa* and *B. oleracea*. 

These results further confirmed the genome duplication events in *B. napus* speciation. It was worth noting that AtJAZ10 had five copies in both *B. rapa* and *B. oleracea*, and 10 copies in rapeseed. However, AtJAZ4 did not have a homologue in *B. rapa* or *B. oleracea*, which meant that AtJAZ4 was the unique gene of *Arabidopsis*. Moreover, AtJAZ7 only had one homologue in *B. oleracea* and no homologue in *B. rapa*. Correspondingly, AtJAZ11 only had one homologue in *B. rapa* and no homologue in *B. oleracea*. It was speculated that these two genes might be lost when *B. rapa* or *B. oleracea* speciation. In addition, AtJAZ12 only had one copy in both *B. rapa* and *B. oleracea*, which implied that AtJAZ12 was not expanded. The above results implied that the different *JAZ* genes/clades might be divisional during the formation and evolution of *Brassica* species.

### 2.3. Conserved Motifs and Gene Structure Analysis Displayed That the JAZ Proteins Clustered in the Same Group Shared Similar Motif Composition and Distribution Order

**Figure 2:**
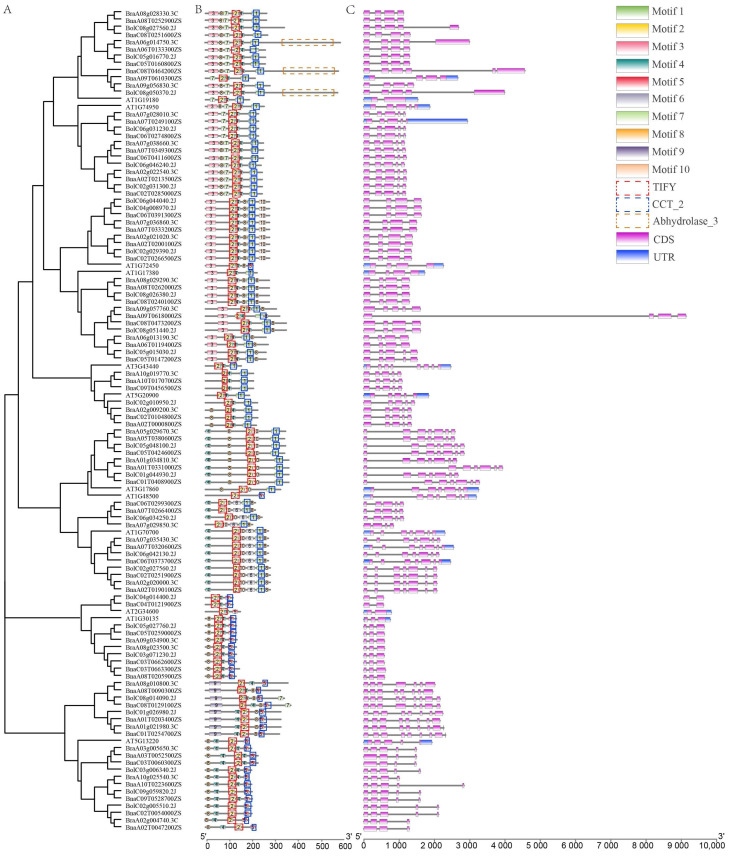
Phylogenetic analysis (**A**), Conserved motif (**B**) and gene structure (**C**) of 114 JAZ proteins from *Arabidopsis*, *B. napus*, *B. rapa*, and *B. oleracea*.

The 102 JAZ proteins were typical and contained both the Jas domain and ZIM domain. Based on that, BnaA06T0133300ZS and BnaA09T0610300ZS had Abhydrolase_3 domains at their C-terminal as well. Conserved motifs analysis showed that 10 conserved motifs were detected, and motifs 2 and 4 existed in all the JAZs. Therefore, it was speculated that sequences of motifs 2 and 4 encoded the conserved functional domain of JAZ protein. However, the distribution of motifs 2 and 4 was variable in different JAZs (Figure 2A). In addition, motif 6 was found only in a branch (homologous to AtJAZ9) of group III. Meanwhile, motif 9 was found only in a branch of group II. Notably, besides all the members of group VI, motif 7 was also presented in BolC08g014090.2J and BnaC08T0129100ZS belonging to group II. Similarly, besides all the members of group III, motif 10 was also found in a branch (homologous to AtJAZ6) of group V. On the whole, the JAZ proteins, clustered in the same group, shared similar motif composition and distribution order. For example, group VI, including the maximal JAZ members, had a conserved “motif 7-2-4-1” and a variable motif 3 or 8. The results showed that the ZIM domain is mostly located in motif 2, and the Jas domain is mostly located in motif 1 or 5. In more detail, the Jas domains of cluster I (group III, IV, V, and VI) were located in motif 1. Instead, the Jas domains of cluster II (group I and II) were located in motif 5. Based on that, it was speculated that the functional difference between clusters I and II might be caused by the difference between motifs 1 and 5. Gene structure displayed that the number of exons and introns of all the JAZ genes was varied from 2 to 9 and 1 to 8, respectively (Figure 2B). It could be found that group I had the least exons (2–3), and the exon numbers of group II had the widest range of variation (3–9).

### 2.4. Chromosomal Location and Expression Pattern of JAZ Genes in B. napus under Multiple Treatments

**Figure 3:**
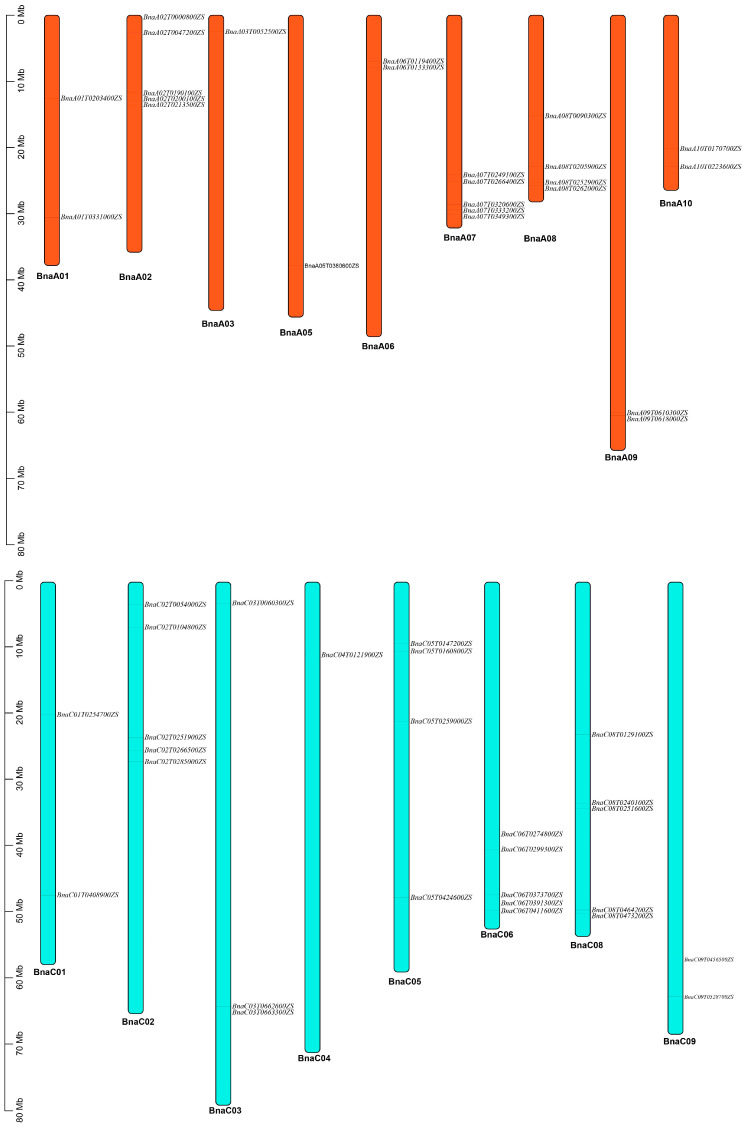
Chromosomal mapping of 51 *BnaJAZs*. The length of the chromosome is represented in Mb. The chromosome number is presented on the bottom of each chromosome, and the names of *BnaJAZs* were at their right.

**Figure 4:**
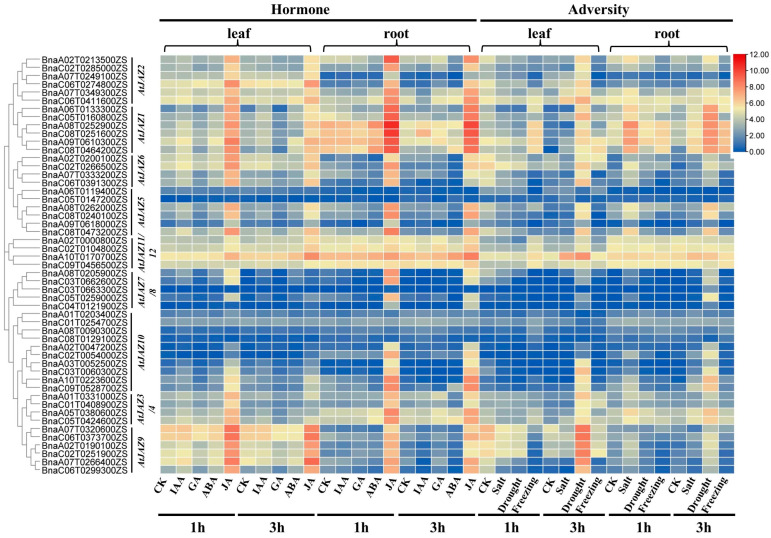
Expression patterns of 51 BnaJAZs under abiotic stress. The expression level of *BnaJAZs* in leaves and roots after 1 h and 3 h of treatment with various hormones and adversities. The homologous genes of *BnaJAZs* in *Arabidopsis* are displayed at the right of *BnaJAZs*. The type and time of treatments are shown at the bottom of the expressional heat map. The tissues of the samples were on the top of the expressional heat map.

All the 51 *BnaJAZs* were assigned to seventeen chromosomes in *B. napus* except for A04 and C07, 24 of which were located in the A sub-genome and 27 in the C sub-genome (Figure 3). The results displayed that the distribution of *BnaJAZs* on each chromosome was uneven. The most *BnaJAZs* (five genes) were present on the chromosomes A02, A07, C02, C06 and C08. Based on the gene expression data from BnIR (http://yanglab.hzau.edu.cn/, accessed on 18 August 2022), it was displayed the expression change of *JAZ* genes in ZS11 under hormone treatment, including IAA, GA, ABA, JA and adversity treatments, including salt, drought and freezing. After IAA, GA, and ABA treatment, the expression level of all JAZs did not present significant changes in the roots and leaves compared with the control. After JA treatment, the homologous genes of AtJAZ1/2/6/9 in *B. napus* showed significantly up-regulated expression in both roots and leaves, while homologous genes of other *AtJAZs* showed certain differentiation on expression level. The homologous genes of *AtJAZ7/8/10* were unchanged in leaves, but some were up-regulated in the roots. About seven *BnaJAZs* were unchanged in both the roots and leaves. Furthermore, we analyzed the expression level of all 51 *BnaJAZs* under abiotic stress. The results showed that the homologue of AtJAZ1 in *B. napus* showed a response to salt, drought and freeze damage stress, especially in roots. However, the homologue of *AtJAZ9* was strongly up-regulated only in leaves after 3 h under drought stress (Figure 4).

### 2.5. Haplotype Analysis of SNPs and SVs Reveals BnaJAZ8.C03 Associated with the Resistance to P. brassicae

**Figure 5:**
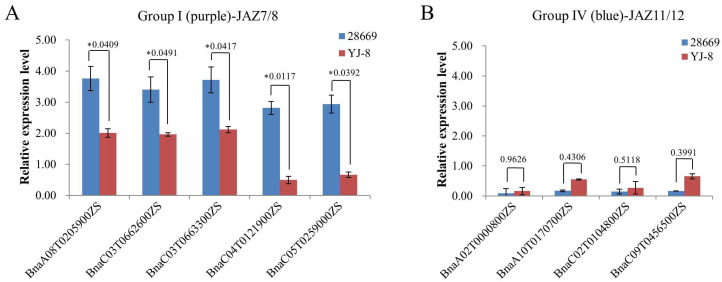
Expression profiles of *BnaJAZs* belonging to group I (**A**) and group IV (**B**) after infection of *P. brassicae*. The columns present the relative expressional foldchange compared with the mock values in the root of resistant and susceptible accessions. The relative expression levels were analyzed by qRT-PCR. The characters on the upper panel were presented with the *p*-values of a *t*-test. * presented the significant difference at a 95% confidence interval.

**Figure 6:**
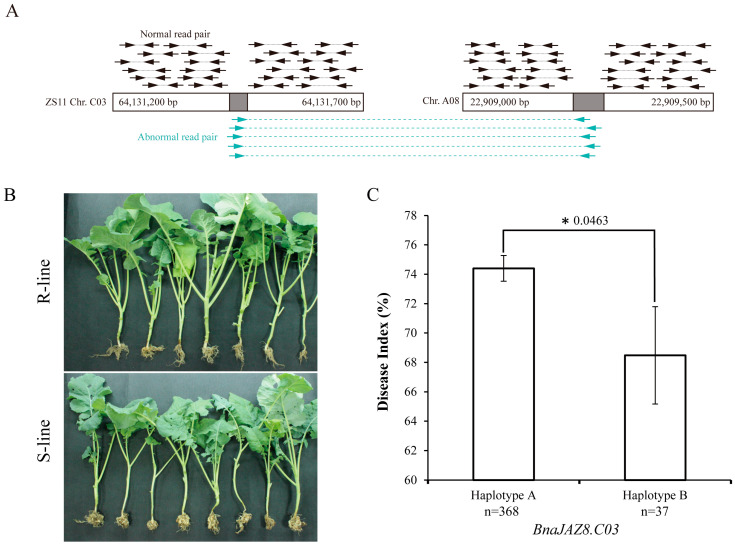
Structural variation and analysis of significance of the differences in resistance. The translocation was detected in *BnaJAZ8.C03*, which resulted in the differences in resistance to *P. brassicae*. The black opposite arrows represent the normal read pairs. The green opposite arrows represent the abnormal read pairs, one end of which was located on the BnaC03 chromosome, but the other end was derived from BnaA08 (**A**). The performance of resistant (“28669”) and susceptible (“YJ-8”) accessions after 42 days infected by *P. brassicae* (**B**). The significance of resistance difference between accessions with and without the translocation in population. Haplotype A represented the average disease index of accessions without the translocation (including 368 accessions), and the haplotype B represented the average disease index of accessions with translocation (including 37 accessions). The *p*-value of the *t*-test is followed by the asterisk (**C**).

To explore the response of the JAZ family to *P. brassicae*, we conducted qRT-PCR to analyze the difference in *JAZ* gene expression between inoculated and un-inoculated by *P. brassicae* in resistant (“28669”) and susceptible (YJ8) line. The results showed that there were no patterns in the expression of *BnaJAZs* in groups II, III, V, and VI. Meanwhile, the expression differential fold of these genes between inoculation and un-inoculation was small, except for an obvious differential expressed gene (DEG), *BnaA02T0200100ZS*, homologous to *AtJAZ6* in group V (Figure S1). It was up-regulated fivefold in “28669” compared with the control, while the change was less than 1.5-fold in YJ8. In addition, five *BnaJAZs* belonging to group I (homologous to *AtJAZ7*/*8*) showed obvious up-regulation in both resistant and susceptible lines. The degree of up-regulation in resistant was higher than in susceptible (Figure 5A). Likewise, the expression of *BnaJAZs* belonging to group IV (homologous to *AtJAZ11*/*12*) was up-regulated, but the degree of which in susceptible was higher than that in resistant (Figure 5B). Because the expression differential fold of *BnaJAZs* belonging to group IV between inoculation and un-inoculation was very small, we further performed the SNPs and SVs detection only in *BnaJAZs* of group I and *BnaA02T0200100ZS* using the whole genome re-sequencing data including 418 rapeseed accessions [30]. A total of 17 SNPs were detected in four *BnaJAZs,* and three SVs were detected in two *BnaJAZs*. Among them, the *BnaA02T0200100ZS* possessed the maximal SNPs (10), and the *BnaC03T0663300ZS* had the maximal SVs (2). In conjunction with this population’s resistance to *P. brassicae*, we further analyzed whether the SNPs or SVs of DEGs affected the resistance to *P. brassicae*. The results showed no significant differences in resistance to *P. brassicae* among different haplotypes divided by SNPs in all the above *BnaJAZs*. After mapping the re-sequence data by pair-end sequencing through an Illumina HiSeq 4000 to the reference genome ZS11, the gray bars represent that normal reads which paired mapped to the *BnaC03T0663300ZS* (*BnaJAZ8.C03*, homologous to *AtJAZ8*) region (Figure S2). While the peak green bars represent the abnormal reads, one end was not mapped to the *BnaC03T0663300ZS* region of chrC03, but to the chrA08 region (Figure 6A). Based on the pair-end reads mapping position of the *BnaC03T0663300ZS* region in the natural population containing 418 accessions, the accessions can be divided into two groups, one containing this translocation and the other not containing the translocation at the *BnaC03T0663300ZS* region (Figure 6A and Figure S2).

The phenotypes of resistant and susceptible lines are shown in Figure 6B. There were 368 accessions that did not contain this translocation, and 37 accessions contained this translocation in the population. Thirteen accessions have not acquired the phenotype. The average disease indexes of accessions with and without this translocation were 74.4%, and 68.5%, respectively. Compared with the accessions without this translocation, the average disease indexes of accessions with this translocation had an 8% reduction. The *t*-test showed that the difference reached a significant level (Figure 6C). The above results implied that the 64 bp translocation in *BnaJAZ8.C03* could significantly change resistance, which may involve the resistance to *P. brassicae* in *B. napus*.

### 2.6. Interaction Network of Key JAZs Identified Their Functional Partner in B. napus

**Figure 7:**
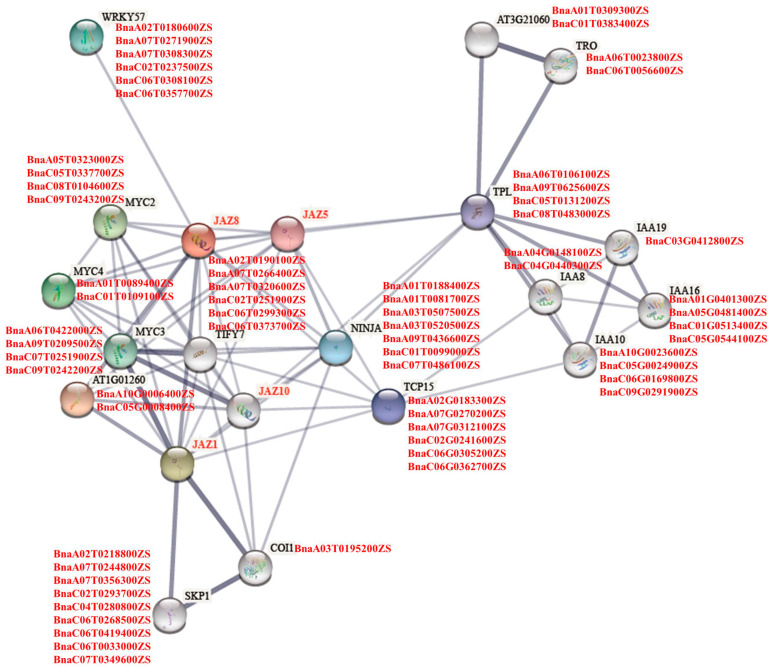
Interaction network of target JAZ proteins. Network nodes represent proteins interactive with JAZ8 directly or indirectly in *Arabidopsis*. Edges present protein–protein associations. The red gene names at the side of the interactive proteins were the homologous genes of interactive proteins in *B. napus*.

Based on the related proteins in *Arabidopsis*, we predicted the potential proteins interacting with JAZ8 using the STRING database. A total of 21 proteins were detected in the PPI network, including four JAZs, which proved that JAZ8 might interact with some JAZ proteins, such as JAZ1/5/10. In addition to some well-known important components of the JAs signaling pathway, such as COI, NINJA, MYC, bHLH and TPL, other proteins have also been identified, such as IAAs, WRKY57, TCP15, SKP1, TRO and so on (Figure 7). It has been reported that WRKY57 affected the expression of JA ZIM-Domain genes transcriptionally to compromise *Botrytis cinerea* resistance. The overexpression of *AtWRKY57* could enhance the drought tolerance in *Arabidopsis* and rice [25,31]. WRKY57 have also been reported to participate in the suppression of abscisic acid at the early infection stage of *Verticillium longisporum* in *B. napus* [32].

Furthermore, SKP1, a core component of the SCF family of E3 ubiquitin ligases, was also detected, which provides specificity in binding to ubiquitin ligase substrate proteins. Studies showed that the SKP1-like protein could influence the resistance to powdery mildew fungus by controlling the abundance of the susceptibility factor RACB in barley [33]. Notably, four IAAs (IAA8/10/16/19) were predicted in this interaction network, suggesting that besides the JA signal transduction pathway, JAZ is also involved in IAA biosynthesis or signal transduction. Based on the homologous collinearity between *B. napus* and *Arabidopsis*, 65 genes were identified that likely took part in the JAZ8 regulation of clubroot resistance in *B. napus* in addition to *BnaJAZs*.

## 3. Discussion

JAZ proteins have been reported to participate in the regulation of stress responses in plants. Comprehensive studies of *JAZ* gene family have been carried out in maize [20], tea plants [34], cotton [35] and other plant species. Most of the time, *JAZ*, as a subfamily, was mentioned in *TIFY* gene family research on rice [18], wheat [36], grape [37], soybean [38], cucumber [39] and so on. In this study, we only focused on the *JAZ* family. There were 51, 25 and 26 *JAZ* genes identified in *B. napus*, *B. rapa* and *B. oleracea*, respectively. This result is inconsistent with previous reports, in which 52, 26, and 27 *JAZ* genes have been reported in *B. napus*, *B. rapa* and *B. oleracea* [40]. The 22 and 21 *JAZ* genes have also been reported in *B. oleracea* [41] and *B. rapa* [42]. We considered that this inconsistency might be due to the difference in the reference genome. On the other hand, the divergent conserved domain formed the non-typical TIFY-JAZ, which also could result in this difference.

In general, the result of the phylogenetic tree was similar to the previous study based on the *AtJAZs* [43]. Because *B. napus* was generated 5000 to 10,000 years ago by the natural hybridization of its two progenitor diploids, *B. rapa* and *B. oleracea*, *B. napus* was an ideal model species to study the evolution of allopolyploid. Moreover, *B. rapa* and *B. oleracea* were produced by extensive triplication of their ancestral species at the genomic level. The three noted species are believed to share a common ancestor with *Arabidopsis*. Therefore, one gene in *Arabidopsis* had an average of 4–6 copies in rapeseed. It could be that most orthologous gene pairs in *B. rapa* and *B. oleracea* remained as homologous pairs in *B. napus* from the tree [44]. In addition, the phylogenetic tree showed the triplication events of the *Brassica* species in the evolution of *JAZ* gene family to a certain extent. Some of the *AtJAZs* had three homologues in the Ar or Co genomes, and some *AtJAZs* experienced expansion or loss after triplication.

Genomic structural variations (SVs) refer to DNA genetic variation over 50 bp in length and are comprised of insertion/deletion (InDel), duplication, inversion and translocation. Numerous studies to identify genes associated with important traits were based on single nucleotide polymorphism (SNP) data, while the SVs were ignored. The accuracy of SVs could be effectively improved by integrating the results of four software SV detection. It laid a foundation for subsequent research. However, the accuracy of SVs needs a further increase compared with the SNPs by improving the sequencing technology and bioinformatics computing methods. SVs not only lead to the fusion of different genes and generate new genes but also play a decisive role in the genetic domestication of crops and the regulation of agronomic trait genes in maize [45], soybeans [46], tomatoes [47], peaches [48,49,50] and other crops. 

In this study, a translocation of a key JAZ, *BnaC03T0663300ZS*, was detected to be associated with the resistance to *P. brassicae* in *B. napus*. This gene was homologous to the *AtJAZ8*, which has been reported to participate in the signaling response during *Agrobacterium* infection to *Arabidopsis*. The overexpression of *AtJAZ8* in *Arabidopsis* could inhibit the activity of VirE3 as a plant transcriptional regulator, activating *AtPR1* gene expression and repressing the expression of *AtPDF1.2*, which significantly decreased the numbers of tumors formed [51]. In addition, *BnaA02T0200100ZS* was screened through the differential expression analysis, although no SV was detected. *BnaA02T0200100ZS* was homologous to *AtJAZ6*, reported previously, and played a crucial role in the rhythmic susceptibility of *Arabidopsis* to *B. cinerea* [52]. 

Based on the above research, we identified 65 proteins that interact with JAZ8 directly or indirectly in *B. napus* through protein–protein interaction analysis and homologous colinearity. Some genes have been reported to participate in Jas’ signaling pathways; others have also been reported to be involved in regulating host resistance to other diseases. The above information could provide some clues for understanding the resistance mechanism of *P. brassicae* in *B. napus*.

## 4. Materials and Methods

### 4.1. Plant Materials, Sequencing and Phenotype Data

This study obtained the seeds of 418 rapeseed accessions from the National Mid-term Gene Bank of Oil Crops Research Institute, Chinese Academy of Agriculture Sciences (OCRI, CAAS). The re-sequencing data of 418 accessions were downloaded from our previous study [30]. The resistance to *P. brassicae* data of 418 accessions was collected by artificial inoculation in 2015 (China, Shenyang) [53]. Among the accessions, we identified a resistant accession, “28669”, and a susceptible accession “, YJ-8”, which came from the National Mid-term Gene Bank of OCRI, CAAS. The total RNA of the root tissue 60 h post-inoculation by *P. brassicae* of “28669” and “YJ-8” were extracted to conduct the qRT-PCR [29]. The *B. napus*, *B. oleracea*, and *B. rapa* materials for sequence analyses were cv. ZS11 (V1.0) [54], JZS (V2.0) [55], and Chiifu (V3.0) [56], respectively.

### 4.2. Identification of the JAZs in B. napus, B. rapa and B. oleracea

To identify the JAZ family members, the genomic sequence of the *B. napus* cv. ZS11 HZAU V1.0 [54], *B. oleracea* cv. JZS V2.0 [55] and *B. rapa* cv. Chiifu V3.0 [56] were downloaded from the website http://www.brassicadb.cn/#/Download/, accessed on 28 April 2022. Twelve *Arabidopsis* typical TIFY-JAZ protein sequences were obtained from TAIR (https://www.arabidopsis.org/, accessed on 28 April 2022), which were used as the queries for searching the JAZ proteins in *B. napus*, *B. rapa* and *B. oleracea* by BLASTP (E-value < 1 × 10^−6^) using TBtools [57]. Meanwhile, the Hidden Markov Model (HMM) profiles of the Jas domain (PF09425) and ZIM domain (PF06200) were downloaded from Pfam (http://pfam.xfam.org/, accessed on 23 May 2022), which were used for protein screening. The conserved domains of candidate JAZs were further confirmed using NCBI-CDD (https://www.ncbi.nlm.nih.gov/Structure/cdd/wrpsb.cgi, accessed on 24 May 2022) and SMART (http://smart.embl-heidelberg.de/, accessed on 24 May 2022). Furthermore, the molecular weight (Mw) and theoretical isoelectric point (pI) of certain JAZ proteins were analyzed in ExPASy (https://web.expasy.org/compute_pi/, accessed on 11 June 2022). Moreover, the subcellular locations of all the JAZ proteins were predicted using ProtComp9.0 from Softberry (http://www.softberry.com/, accessed on 11 June 2022).

### 4.3. Phylogenetic, Gene Structure and Conserved Motif Analyses

To explore the evolutionary characteristics of JAZs from *Arabidopsis*, *B. napus*, *B. rapa* and *B. oleracea*, multiple JAZ protein sequences were aligned by MUSLE [58]. Then, the alignment results were used for constructing an un-rooted neighbor-joining (NJ) phylogenetic tree using MEGA 11 software [59] with 1000 bootstrap replicates. Finally, we beautified the phylogenetic tree in iTOL (https://itol.embl.de/, accessed on 26 May 2022). The conserved motifs were searched in MEME (https://meme-suite.org/meme/tools/meme, accessed on 27 May 2022), and the detection parameter of conserved motifs was set to 10 for fear of missing some rare motifs that may have specific functions. TBtools software was used for drawing the exons, introns, conserved domain coverage and motif distribution.

### 4.4. Chromosomal Location and Expression Pattern Analysis of JAZ Genes in B. napus under Multiple Treatment

Based on the genomic sequence annotation file of *B. napus* cv. ZS11 obtained from the BnPIR database (http://cbi.hzau.edu.cn/bnapus/, accessed on 28 Aprial 2022), the chromosomal positions of all the *BnaJAZs* were drawn using TBtools. The expression data of *BnaJAZs* in leaf and root under hormone and adversity treatment were acquired from BnIR (http://yanglab.hzau.edu.cn/BnIR, accessed on 18 August 2022). The qRT-PCR results analysis was performed on LightCycler 480 SYBR Green I Mastermix, and a LightCycler 480II real-time PCR system (Roche, Switzerland). The transcript abundance was calculated from three biological and three technical replicates with *Bna.Actin7* as an internal control. The fold-change was estimated using the 2^−ΔΔCt^ [60]. The gene-specific primer sequences (Table S1) and heat maps were also designed and drawn with TBtools.

### 4.5. Structural Variation Detection, Resistance Difference Analysis and Interaction Network Construction of Target Genes

Based on the re-sequencing data [30], we carried out the population’s SNPs and structural variation (SVs) detection. Therein, the SVs were detected by using three tools, LUMPY 0.2.13 [61], Manta 1.6.0 [62], and smoove 0.2.8 (https://github.com/brentp/smoove, accessed on 20 August 2022). To improve the accuracy, the variants detected by at least two tools were kept using SURVIVOR 1.0.6 [63]. SPASS software detected the significance of the resistance differences among different haplotypes divided by SNPs or SVs in each target gene. The interaction networks of the target JAZs were generated using the STRING database (https://cn.string-db.org/, accessed on 24 August 2022).

## Data Availability

Not applicable.

## Supplementary Materials

The following supporting information can be downloaded at: https://www.mdpi.com/article/10.3390/ijms232112862/s1. Figure S1: Expression profiles of *BnaJAZs* belonging to orange, green, red and yellow groups divided by phylogenetic analysis in the roots of resistant and susceptible accessions after 60 h infected by *P. brassicae*; Figure S2: The differential performance of reads in *BnaC03T0663300ZS* (*BnaJAZ8.C03*) between resistant and susceptible accessions; Table S1: Gene-specific primers sequence of qRT-PCR; Table S2: Features of 102 JAZ proteins in *B. rapa*, *B. oleracea* and *B. napus*.
