# Supplementary material for: Genome-Wide Identification and Variation Analysis of JAZ Family Reveals BnaJAZ8.C03 Involved in the Resistance to Plasmodiophora brassicae in Brassica napus
Source: Int J Mol Sci. 2022 Oct 25;23(21):12862. doi: 10.3390/ijms232112862 (PMC9657359; doi:10.3390/ijms232112862)
Supplement: Supplementary file 1 [file ijms-23-12862-s001.zip › Table S and Figure S figure legends20221024.pdf]

Genome-wide identification and variation analysis of *JAZ* family reveals *BnaJAZ8.C03* involved in the resistance to *Plasmodiophora brassicae* in *Brassica napus*

Lixia Li[1, #], Gaoxiang Ji[1, #], Wenjie Guan[1], Fang Qian[1], Hao Li[1], Guangqin Cai[1,*] and Xiaoming Wu[1,*]

1 Key Laboratory of Biology and Genetic Improvement of Oil Crops, Ministry of Agriculture and Rural Affairs, Oil Crop Research Institute, Chinese Academy of Agricultural Sciences, Hubei, China

# Li Lixia and Ji Gaoxiang are contributed equally in this study

* Correspondence: caiguangqin@caas.cn; Tel.: +86 27 86711659; wuxm@oilcrops.cn; Tel.: +86 27 86812906

Table S1 The gene-specific primers sequence of qRT-PCR

Gene ID	Forward	Reverse
BnaA01T0203400ZS	CGTCGACTGAGCCAAGCA	TCTCCTGAGGGAAGGGCC
BnaA01T0331000ZS	CCTGAAAAGGCCAAGGCG	CGGAGGCACGAGCATGAT
BnaA02T0000800ZS	ACGGACGCTCCTTCCTCT	GTGGCAGCAGCAGCAATG
BnaA02T0047200ZS	CCGTTCCGAGTCATCCACC	AGGCACGTGGACAGGAGA
BnaA02T0190100ZS	GCTGCGGCATCATCAAGC	CAGGACCTGCTGCGTTGA
BnaA02T0200100ZS	CCAACGAGTTCCGGGACC	AGCTCTAGCCACAGCCCT
BnaA02T0213500ZS	GTGTCTCGACAGCCAACGA	CCTTGGCTCCAGCGGAAG
BnaA03T0052500ZS	ACAATGGAACCGTCTCCGT	CCTTAGGGTGGTCGGAGGA
BnaA05T0380600ZS	CCTGATAAGGCCAAGGCGA	GCATGATGGGCCACCTGT
BnaA06T0119400ZS	ATTCGGATCGCGTGCTGT	GCTTTGCCTGGAGACGGT
BnaA06T0133300ZS	AAGCCTGAGACCAACGGC	GGCCATGGAACCCACGTT
BnaA07T0249100ZS	TCCCAACGGCTTCACAGC	TTGGCTCTTGAGCTGCGG
BnaA07T0266400ZS	TCCAGCACGAAAGCAGCT	AACGCGCCGCTATACTGG
BnaA07T0320600ZS	AGCCCGACGTTGACGATG	TACTGGCCGCGGAAACTG
BnaA07T0333200ZS	AGGGCTGTGGCTAGAGCT	TGCAGGCTGTCGTGAGTG
BnaA07T0349300ZS	GTCAGCAGCCGACGATGA	GGCTCCAGAGGACGAGGA
BnaA08T0090300ZS	CCAGAAGCCCGGCAGAAA	GGGTTGGCTGCAAAGTGC
BnaA08T0205900ZS	GGGTCGGATCCTCGGAAC	TCGTCGTGAATGCTGATGGT
BnaA08T0252900ZS	AGCCGAGCTTCTCCCTGA	TGGTCGTTGTGGGCTGAC
BnaA08T0262000ZS	CCAACTTCACCCGGCGAT	GGACCAGGCTTTCGGACC
BnaA09T0610300ZS	TGGCGGGCAAGTGATTGT	CGGTTCCTTTGCTCGCCA
BnaA09T0618000ZS	CGCCGGAGAAGTCCAGTT	CGAGGGCTTTGGTCTCCG
BnaA10T0170700ZS	GCCGGAGAAGGTGCTGAG	GGACGGGTCGTTGAAGCA

BnaA10T0223600ZS	CGCGGTCGTAGTTTCCGA	CGGAAGGGTTAGCACCAGT
BnaC01T0254700ZS	CAGGCGGTCCAGCAAGTT	TGCTTGGCTCAGTCGACG
BnaC01T0408900ZS	CCTGAAAAGGCCAAGGCG	AATCAACGGAGGCACGGG
BnaC02T0054000ZS	CCGTTCCGAGTCATCCACC	AGGCACGTGGACAGGAGA
BnaC02T0104800ZS	ACGGACGCTCCTTCCTCT	GTGGCAGCAGCAGCAATG
BnaC02T0251900ZS	GCTGCGGCATCATCAAGC	CAGGACCTGCTGCGTTGA
BnaC02T0266500ZS	AGGGCTGTGGCTAGAGCT	GGAGTCTCAGGCTGTCGC
BnaC02T0285000ZS	GTGTCTCGACAGCCAACGA	TCCCTTGGCTCCAGAGGA
BnaC03T0060300ZS	CGGTCTCGAGAAGAAGCAGA	ACCTCGGAAACTGCGACG
BnaC03T0662600ZS	CGGAACAGGTTGACCCGA	TCGTCGTGAATGCTGATGGT
BnaC03T0663300ZS	CGGAACAGGTTGACCCGA	TGGTACGGTGAAGCTGCT
BnaC04T0121900ZS	CCCATCTCCAGGCAAAAGC	AGGATCCGAGCCGTCTGA
BnaC05T0147200ZS	ATTCGGATCGCGTGCTGT	GCTTTGCCTGGAGACGGT
BnaC05T0160800ZS	TTCTACGGCGGGCAAGTG	TGGGGTAGGAGCGATGCT
BnaC05T0259000ZS	CGAAACAGGTCGACCCGA	TCGTCGTGAATGCTGATGGT
BnaC05T0424600ZS	GGGAGCCAGGATGTTCCC	TGAGACTGGAGACCCGGC
BnaC06T0274800ZS	CGCGATGACAAGCAAGCC	ACCTCTTCCTTGGCCCCA
BnaC06T0299300ZS	TGGGCTTCAGCGACAAGC	ACGCACCGCTATACTGGC
BnaC06T0373700ZS	TGCTGGGAACGGTTTGCA	GCGCTAGTGGCAGAGCTT
BnaC06T0391300ZS	AGGGCTGTGGCTAGAGCT	TGCAGGCTGTTGTGAGGG
BnaC06T0411600ZS	AAGCCGAGCTTCTCGCAG	TCATCGTCGGCTGCTGAC
BnaC08T0129100ZS	CGGCATCCTCCGCAAGAT	TCGGAGCCGGAGAACTCA
BnaC08T0240100ZS	CCAACTTCACCCGGCGAT	GGACCAGGCTTTCGGACC
BnaC08T0251600ZS	AGCCGAGCTTCTCCCTGA	AGTCGTTGTGGGCTGACG
BnaC08T0464200ZS	CCTCGTGGCCGAAACCTT	CGGCTACGGCTGTATGGG
BnaC08T0473200ZS	GCCGGAGAAGCCCAGTTT	GGCAACCCGAGATCCGAG
BnaC09T0456500ZS	AGGTGGATGCGGTGTTGG	CTCAGCACCTTCTCCGGC
BnaC09T0528700ZS	CTTCTTGACCGCCGTCGT	GGTCGAGGTTTCGGAAGGG

Table S2 Features of 102 JAZ proteins in *B. rapa*, *B. oleracea* and *B. napus*

Protein name	Amino acid	Molecular weight	Theoretical pI	Grand average of hydropathicity	Subcellular localization
BraA01g021980.3C	319	35112.41	8.78	-0.75	Nucleus.
BraA01g034810.3C	353	37579.04	9.3	-0.387	Nucleus.
BraA02g004740.3C	184	20606.73	9.99	-0.642	Nucleus.
BraA02g009200.3C	221	23082.95	4.97	-0.234	Nucleus.
BraA02g020000.3C	274	29342.19	9.86	-0.372	Nucleus.
BraA02g021020.3C	273	30157.81	9.11	-0.836	Nucleus.
BraA02g022540.3C	240	26197.55	9.21	-0.559	Nucleus.
BraA03g005650.3C	195	21818.19	9.91	-0.465	Nucleus.
BraA05g029670.3C	339	36295.69	9.3	-0.422	Nucleus.
BraA06g013190.3C	256	28753.36	9.28	-0.931	Nucleus.
BraA06g014750.3C	573	62802.31	8.69	-0.336	Nucleus.
BraA07g028010.3C	224	24478.56	9.08	-0.667	Nucleus.
BraA07g029850.3C	199	21776.68	9.34	-0.417	Nucleus.
BraA07g035430.3C	267	28812.7	9.76	-0.429	Nucleus.
BraA07g036860.3C	274	30596.33	9.08	-0.883	Nucleus.
BraA07g038660.3C	245	26810.27	9.2	-0.595	Nucleus.
BraA08g010800.3C	349	38488.47	9	-0.59	Nucleus.
BraA08g023500.3C	130	14971.78	9.85	-0.983	Nucleus.
BraA08g028330.3C	259	28310.18	9.49	-0.463	Nucleus.
BraA08g029290.3C	270	30031.67	8.32	-0.934	Nucleus.
BraA09g034900.3C	133	15329.22	9.62	-0.972	Nucleus.
BraA09g056830.3C	273	29810.66	9.49	-0.549	Nucleus.
BraA09g057760.3C	300	33172.7	9.8	-0.896	Nucleus.
BraA10g019770.3C	203	21187.76	6.91	-0.367	Nucleus.
BraA10g025540.3C	184	20581.83	9.95	-0.596	Nucleus.
BolC01g026980.2J	317	34910.06	9.23	-0.847	Nucleus.
BolC01g044930.2J	354	37560.06	9.34	-0.391	Nucleus.
BolC02g005510.2J	196	21800.12	9.96	-0.576	Nucleus.
BolC02g010950.2J	220	23089.94	4.97	-0.234	Nucleus.
BolC02g027560.2J	274	29540.52	9.93	-0.403	Nucleus.
BolC02g029390.2J	272	29893.25	6.67	-0.856	Nucleus.
BolC02g031300.2J	240	26238.56	9.3	-0.581	Nucleus.
BolC03g006340.2J	195	21688	9.95	-0.445	Nucleus.
BolC03g071230.2J	130	14971.78	9.85	-0.983	Nucleus.
BolC04g008970.2J	271	30322.98	9.47	-0.968	Nucleus.
BolC04g014400.2J	116	13127.05	9.15	-0.601	Nucleus.
BolC05g015030.2J	256	28397.94	9.69	-0.877	Nucleus.
BolC05g016770.2J	254	27255.92	9.71	-0.474	Nucleus.
BolC05g027760.2J	132	15274.14	9.62	-1.033	Nucleus.
BolC05g048100.2J	340	36284.48	9.47	-0.479	Nucleus.

BolC06g031230.2J	224	24491.61	9.23	-0.609	Nucleus.
BolC06g034250.2J	239	26175.69	8.82	-0.395	Nucleus.
BolC06g042130.2J	266	28723.6	9.71	-0.415	Nucleus.
BolC06g044040.2J	271	30322.98	9.47	-0.968	Nucleus.
BolC06g046240.2J	235	26035.25	9.3	-0.714	Nucleus.
BolC08g014090.2J	334	36800.06	8.74	-0.831	Nucleus.
BolC08g026380.2J	268	29748.45	8.84	-0.918	Nucleus.
BolC08g027560.2J	334	36942.02	9.6	-0.546	Nucleus.
BolC08g050370.2J	560	61991.37	9.35	-0.386	Nucleus.
BolC08g051440.2J	343	37966.61	9.39	-0.965	Nucleus.
BolC09g059820.2J	197	21998.47	10.07	-0.549	Nucleus.
BnaC05T0147200ZS	256	28397.94	9.69	-0.877	Nucleus.
BnaC08T0240100ZS	268	29788.51	8.84	-0.929	Nucleus.
BnaA06T0119400ZS	266	29804.4	9.18	-0.938	Nucleus.
BnaA08T0262000ZS	267	29706.46	9.11	-0.948	Nucleus.
BnaC08T0473200ZS	343	38001.73	9.49	-0.995	Nucleus.
BnaA07T0333200ZS	274	30596.33	9.08	-0.883	Nucleus.
BnaC06T0391300ZS	271	30322.98	9.47	-0.968	Nucleus.
BnaC02T0266500ZS	272	29949.36	7.07	-0.85	Nucleus.
BnaA02T0200100ZS	273	30161.8	9.11	-0.833	Nucleus.
BnaA09T0618000ZS	315	35081.54	9.67	-1.003	Nucleus.
BnaA07T0249100ZS	224	24585.7	9.14	-0.659	Nucleus.
BnaA09T0610300ZS	211	23030.09	9.42	-0.494	Nucleus.
BnaC06T0274800ZS	224	24461.58	9.23	-0.607	Nucleus.
BnaA02T0213500ZS	240	26197.55	9.21	-0.559	Nucleus.
BnaC05T0160800ZS	254	27255.92	9.71	-0.474	Nucleus.
BnaC02T0285000ZS	240	26203.56	9.3	-0.577	Nucleus.
BnaC08T0251600ZS	264	28742.64	9.65	-0.469	Nucleus.
BnaA06T0133300ZS	254	27300.06	9.85	-0.484	Nucleus.
BnaC08T0464200ZS	564	62217.59	9.18	-0.373	Nucleus.
BnaA08T0252900ZS	259	28310.18	9.49	-0.463	Nucleus.
BnaA07T0349300ZS	245	26757.17	9.2	-0.603	Nucleus.
BnaC06T0411600ZS	246	27237.72	8.96	-0.617	Nucleus.
BnaC03T0662600ZS	130	14971.78	9.85	-0.983	Nucleus.
BnaA08T0205900ZS	130	14971.78	9.85	-0.983	Nucleus.
BnaC03T0663300ZS	144	16741.87	8.75	-0.651	Nucleus.
BnaC05T0259000ZS	132	15241.15	9.47	-0.971	Nucleus.
BnaC04T0121900ZS	116	13127.05	9.15	-0.601	Nucleus.
BnaC05T0424600ZS	335	35762.95	9.47	-0.493	Nucleus.
BnaA05T0380600ZS	336	35871.11	9.51	-0.474	Nucleus.
BnaA01T0331000ZS	353	37579.04	9.3	-0.387	Nucleus.
BnaC01T0408900ZS	353	37522.09	9.25	-0.373	Nucleus.
BnaC06T0299300ZS	212	23318.25	9.04	-0.509	Nucleus.
BnaA07T0266400ZS	212	23408.39	9.03	-0.533	Nucleus.

BnaC06T0373700ZS	266	28532.51	9.57	-0.32	Nucleus.
BnaA02T0190100ZS	274	29342.19	9.86	-0.372	Nucleus.
BnaA07T0320600ZS	266	28640.52	9.8	-0.433	Nucleus.
BnaC02T0251900ZS	274	29406.24	9.93	-0.438	Nucleus.
BnaC02T0104800ZS	221	23082.95	4.97	-0.234	Nucleus.
BnaC09T0456500ZS	201	21251.74	6.45	-0.448	Nucleus.
BnaA02T0000800ZS	216	22474.21	4.96	-0.244	Nucleus.
BnaA10T0170700ZS	203	21430.97	6.92	-0.477	Nucleus.
BnaA10T0223600ZS	196	21819.2	9.91	-0.536	Nucleus.
BnaC02T0054000ZS	196	21800.12	9.96	-0.576	Nucleus.
BnaC09T0528700ZS	199	22461.96	9.99	-0.531	Nucleus.
BnaC01T0254700ZS	314	34533.59	8.31	-0.79	Nucleus.
BnaA01T0203400ZS	320	35095.26	9.12	-0.796	Nucleus.
BnaA08T0090300ZS	318	35012.22	9.13	-0.794	Nucleus.
BnaC08T0129100ZS	362	39793.75	8.28	-0.648	Nucleus.
BnaA02T0047200ZS	212	23994.92	9.95	-0.327	Nucleus.
BnaC03T0060300ZS	223	25198.16	9.81	-0.261	Cell membrane. Nucleus.
BnaA03T0052500ZS	223	25247.27	9.81	-0.239	Cell membrane. Chloroplast. Nucleus.

Figure S1 Expression profiles of *BnaJAZs* belonging to group Ⅱ, Ⅲ, Ⅴ and Ⅵ after infection of *P. brassicae*. The column presented the relative expressional fold-change compared with mock in root of resistant and susceptible accessions. The relative expression levels were analyzed by qRT-PCR. The characters on the up panel were presented the p-value of t-test. * was presented the significant difference at a 95% confidence interval.

Figure S2 Differential performance of reads in *BnaJAZ8.C03* between resistant and susceptible accessions. The gray bars represent that normal reads which paired mapped to the *BnaC03T0663300ZS* region. While, the peak green bars represent the abnormal reads, one end of which has not mapped to the *BnaC03T0663300ZS* region of chrC03, but to the chrA08 region

Table S1 Gene-specific primer sequences of qRT-PCR

Table S2 Features of 102 JAZ proteins in *B. rapa*, *B. oleracea* and *B. napus*
