# Supplementary figures and images for: Genome-Wide Identification and Variation Analysis of JAZ Family Reveals BnaJAZ8.C03 Involved in the Resistance to Plasmodiophora brassicae in Brassica napus
Source: Int J Mol Sci. 2022 Oct 25;23(21):12862. doi: 10.3390/ijms232112862 (PMC9657359; doi:10.3390/ijms232112862)

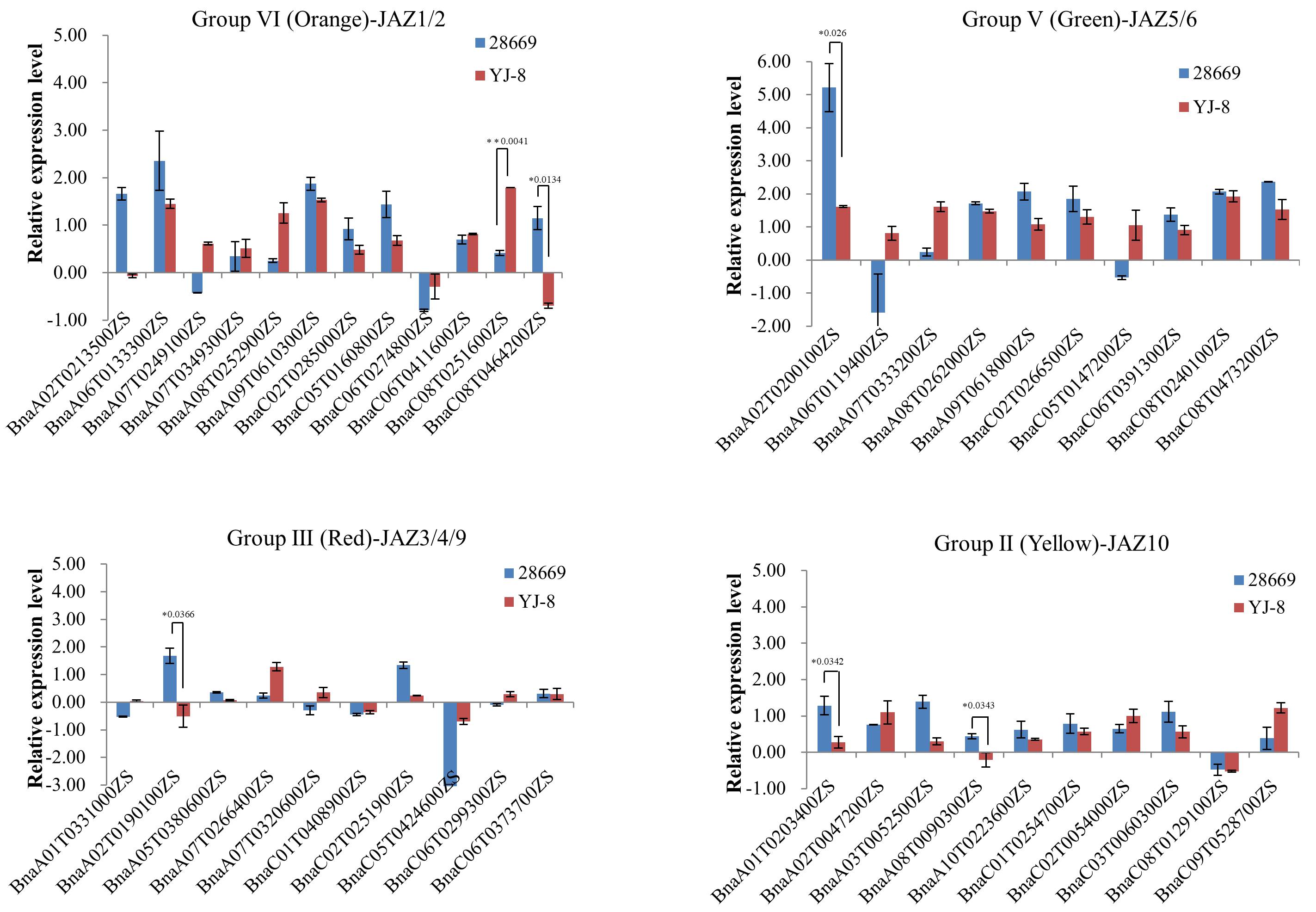

Supplement: Supplementary file 1 [file ijms-23-12862-s001.zip › Figure S1.jpg]
